# Two distinct functions of *Lim1* in the *Drosophila* antenna

**DOI:** 10.17912/micropub.biology.001229

**Published:** 2024-06-17

**Authors:** Dylan M Dolezal, Mei-ling A Joiner, Daniel F Eberl

**Affiliations:** 1 Biology, University of Iowa, Iowa City, Iowa, United States

## Abstract

The *Lim1* transcription factor is required in *Drosophila* for patterning the eye-antennal disk. At the adult stage, *Lim1* is strongly expressed in Johnston’s Organ (JO) neurons, the antennal auditory organ. Using RNAi-mediated knockdown of *Lim1* using a strong neuronal driver, we find a significant reduction in electrophysiological responses to auditory stimuli, recorded from the antennal nerve. This reduction can be accounted for by *Lim1* knockdown in the auditory subset of JO neurons, with no effect of knockdown in JO neuron subsets associated with wind or gravity detection. Conversely, *Lim1* knockdown in JO sense organ precursors had no effect on hearing. Mosaic animals with antennal clones of the *Lim1 ^E9^* null mutation showed morphological defects in the antenna, and significant auditory electrophysiological defects. Our results are consistent with two distinct functions for *Lim1* in the antenna, including an early patterning function in the eye-antennal disk, and a later neural differentiation function in the JO neurons.

**Figure 1. Expression of Lim1 in Drosophila antenna and phenotypes in hearing function:**
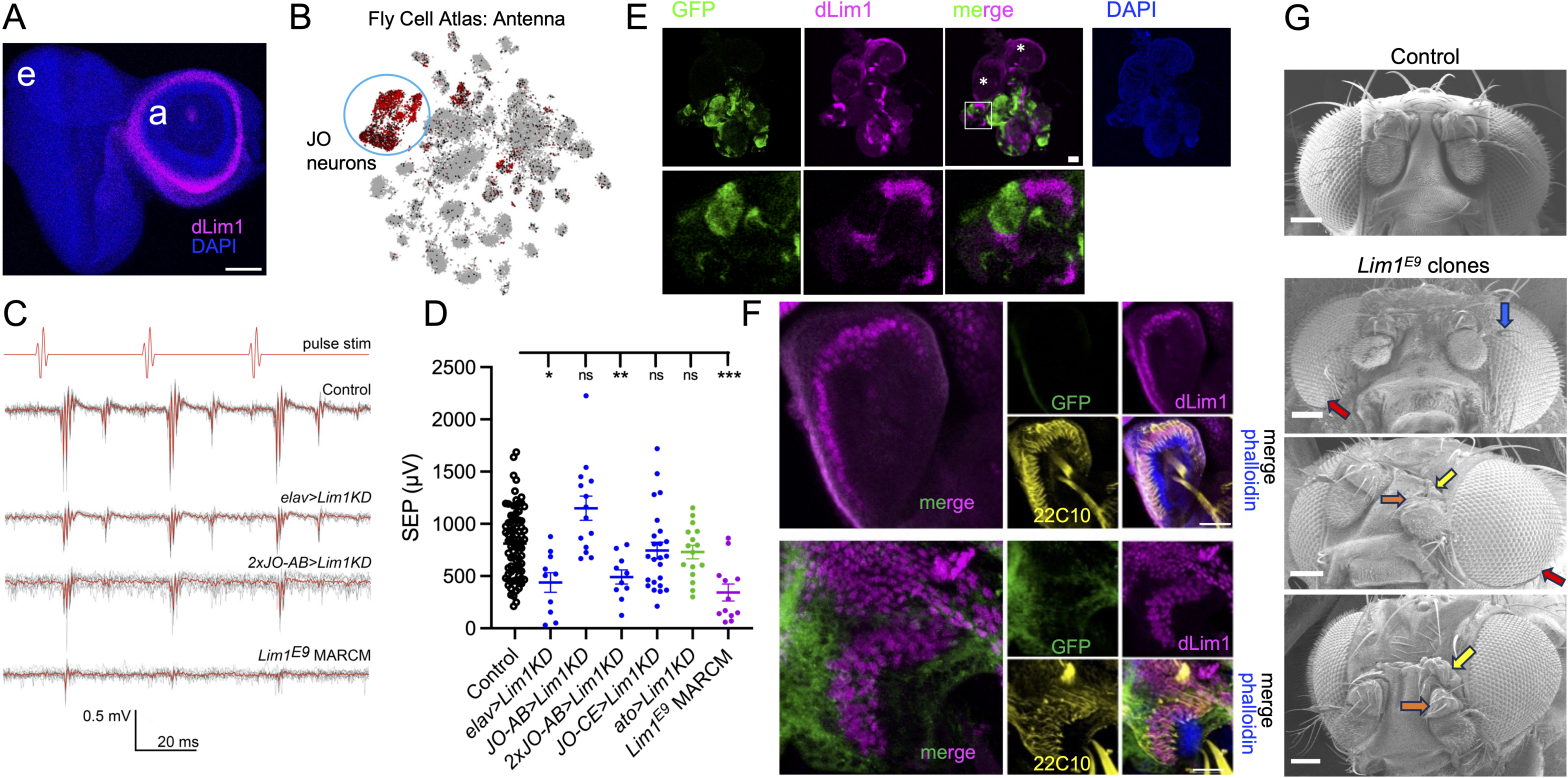
A) Third instar eye-antennal disk stained for Lim1 protein (magenta) shows expression in the second antennal segment. Counterstain: DAPI (blue). e = eye, a = antenna. B) Expression of *Lim1* (red) depicted over Fly Cell Atlas antennal single nucleus RNA sequencing clusters (Li et al 2022) indicates that *Lim1* is expressed primarily in JO neurons (circled). C) Sound-evoked potential (SEP) traces from a control animal ( *elav-Gal4* alone) shows robust response to computer-generated pulse song (pulse stim). A representative *Lim1* knockdown animal ( *elav-Gal4; UAS-Lim1-RNAi* ) shows reduced SEP, as do representative animals with *Lim1* knockdown using *2xJO-AB* driver or *Lim1 ^E9^* clones. D) Scatterplots of SEPs from control and *Lim1* RNAi knockdown animals. Each dot represents the SEP from one antenna. Bars and whiskers represent mean ± standard error of the mean. Statistically significant differences from control are indicated at the top (Welch’s one-way ANOVA, p < 0.0001, Dunnett’s T3 post-hoc multiple comparisons: ns = not significant, * p < 0.05, ** p < 0.01, ***p < 0.001). E) (upper row) Third instar larval imaginal disks and brain of *Lim1 ^E9^* MARCM clone animal stained for the clone marker GFP (green), as well as anti-dLim1 (magenta) and the nuclear stain DAPI (blue). As *ey* -FLP does not express in the wing disks (asterisks), no GFP-positive clones are generated. (lower row) Magnified views of one eye-antennal imaginal disk, highlighted by the box in the upper row. In GFP-positive clones, dLim1 staining is absent. Scale bar in merged panel: 50 μm. F) Pupal antennae from control (upper row) and *Lim1 ^E9^* MARCM clone animals (lower row), stained for GFP (green), anti-dLim1 (magenta) as well as the neuronal marker Futsch (22C10, yellow), and actin (phalloidin, blue). Scale bars in merged images: 10 μm. G) Scanning electron micrographs of heads from a control animal and *Lim1 ^E9^* MARCM clone animals. Examples of defects in clone animals include deformed arista (blue arrow), reduced or deformed second antennal segment (orange arrows), excess tissue dorsal to the antenna (yellow arrows) and reduced eye size, usually on the ventral side (red arrows). Scale bars: 100 μm.

## Description

*Lim1* is a conserved transcription factor gene with two vertebrate paralogs, *Lhx1* and *Lhx5* , that regulate early developmental patterning (reviewed by Yasuoka & Taira, 2021). In *Xenopus* , ectopic co-injection of mRNAs encoding *Xlim1* and the Lim-binding domain protein *XLdb1* (homologous to the *Drosophila* *Chip* gene) can induce a secondary axis in the embryo. Interestingly, co-injection of *Drosophila* *Lim1* mRNA with *XLdb1* into *Xenopus* embryos induces a secondary axis with almost equal efficacy to *Xlim1* (Tsuji et al., 2000) . In *Drosophila* , *Lim1* contributes to patterning of the third instar larval eye-antennal disk (Ku & Sun, 2017; Lilly et al., 1999; Weasner & Kumar, 2013) , where its expression in the antennal domain represses *Eyeless* ( *ey* ) expression from extending from the eye in a *Chip* -dependent manner (Roignant 2010). In the eye-antennal disk, *Lim1* is expressed primarily in the presumptive second antennal segment (Lilly et al., 1999, Figure 1A ) where it is critical for establishing a boundary between the proximal and distal segments (Ku & Sun, 2017) , as well as in a central spot in the disk from which the distal-most antennal fates develop ( (Tsuji et al., 2000) . In the adult *Drosophila* antenna, expression of the single *Lim1* gene is largely restricted to neurons of Johnston’s Organ (JO) (Li et al., 2022, Figure 1B ), responsible for auditory function.

Based on this expression pattern we wanted to investigate the requirement of *Lim1* in auditory function. To accomplish this, we used the *elav-Gal4* driver to target *Lim1* RNAi-mediated knockdown in JO neurons and tested for hearing function by recording sound-evoked potentials (SEPs) from the antennal nerve (Eberl et al., 2000; Eberl & Kernan, 2011) . Presentation of computer-generated pulse song elicited robust SEPs from control flies, while flies with strong *Lim1* knockdown in all neurons with the *elav-Gal4* driver showed reduced SEPs ( Figure 1C,D ) (p = 0.017). Subsets of JO neurons have been identified that serve primarily an auditory function (JO-AB neurons) or primarily wind-gravity functions (JO-CE) (Kamikouchi et al., 2009; Kamikouchi et al., 2006; Yorozu et al., 2009) . Using the driver expressed in the JO-AB neurons, we found no significant effects on hearing ( Figure 1D, p = 0.071). However, to try to match the strong expression of the *elav-Gal4* driver, we doubled the expression in JO-AB neurons using a strain with the *JO-AB* driver inserted both in the second and third chromosomes. With this enhanced expression of *Lim1-RNAi* in JO-AB neurons, we observed a significant reduction in hearing (labeled *2xJO-AB* in Figure 1C,D, p = 0.0046), to a similar level observed with the *elav-Gal4* driver. These results support a functional role for *Lim1* in the neuronal mechanisms of hearing. Expression of *Lim1-RNAi* in JO-CE neurons had no impact on hearing function ( Figure 1D, p = 0.97), consistent with this subset of neurons responding primarily to tonic stimuli (wind and gravity) rather than vibratory (auditory) stimuli (Kamikouchi et al., 2009; Yorozu et al., 2009) . However, we cannot rule out that these JO-CE drivers knock down *Lim1* insufficiently in these neurons, and doubling the expression in JO-CE is currently not possible with available strains.

JO chordotonal precursors are specified by expression of the bHLH proneural transcription factor *atonal* ( *ato* ) (Jarman et al., 1993; Jarman & Groves, 2013) . To determine whether the requirement of *Lim1* in hearing function is related to specification of the JO precursor cells, we used *ato-Gal4* to drive *Lim1-RNAi* . We found no significant effect on hearing ( Figure 1D, p = 0.84) in these animals.

RNAi approaches usually result in only partial knockdown of the target gene mRNAs (Dietzl 2007). Unfortunately, null mutations of *Lim1* are lethal, preventing us from testing hearing function in adults. Therefore, we used the Mosaic Analysis with a Repressible Cell Marker (MARCM) approach (Lee 2001) to generate marked clones of *Lim1 ^E9^* null mutant tissue in the eye-antennal disk using the yeast FLP site-specific recombinase driven by the *ey* promotor. The *ey* gene is expressed at embryonic stages in both eye and antennal primordia before its restriction to only the eye during the second larval instar (Kumar & Moses, 2001) .

*Lim1 ^E9^* mutant clones in late larval eye-antennal disks stained for the clonal marker GFP and for Lim1 show loss of Lim1 staining, while Lim1 staining is retained in the non-mutant tissue lacking the GFP clonal marker ( Figure 1E ). Similarly, in stage P6-P9 pupal antenna, *Lim1* mutant clones lost Lim1 staining ( Figure 1F ). Adult flies with *Lim1 ^E9^* mutant clones displayed a variety of external phenotypes in and near the antenna ( Figure 1G ). These include reduced or deformed antennal structures affecting some or all of the arista, the third antennal segment, and the second antennal segment containing the JO. In addition, other head structures are often affected, including reduction of eye tissue, especially on the ventral side, unusual tissue growths dorsal to the antenna, and missing or misshapen frontal and frontoorbital bristles. These defects are consistent with mutant effects of a different *Lim1* mutation, *Lim1 ^7B2^* (Tsuji et al., 2000) , on antennal development.

To assess impacts on hearing function, we measured SEPs from *Lim1 ^E9^* mutant clone animals and found a significant reduction in hearing compared to controls ( Figure 1D, p = 0.0004). Loss of *Lim1* in MARCM clone animals is mosaic, so in most of these antennae, likely only a subset of neurons lacks *Lim1* , as seen by staining in larval disks and pupal antenna ( Figure 1E,F ). Because of this and the fact that antennae carrying *Lim1* mutant clones are often somewhat deformed, we cannot conclude that the loss of hearing function is solely due to neuronal loss of *Lim1* , but rather may reflect a combination of mechanical disruptions of the antenna and loss of *Lim1* in a subset of JO neurons.

Overall, our results are consistent with two distinct roles for *Lim1* in the *Drosophila* antenna. The early patterning role, which has been previously described to restrict *ey* expression from the antennal region, and to establish segment boundaries, is important for the overall formation of the antenna. The later role, where *Lim1* expression is restricted to the JO neurons, is important for proper physiological differentiation of those neurons for their function in hearing.

## Methods

***Drosophila strains***

*Drosophila melanogaster* strains used in this study included several *Gal4* drivers. *P{GawB}elav ^C155^* (herein referred to as *elav-Gal4* ) expresses strongly in all neurons (Lin & Goodman, 1994) . The *ato-Gal4* driver (Hassan et al., 2000) drives expression in a chordotonal organ pre-neural pattern. To drive expression in the auditory subset of JO neurons, we used *JO15-2-Gal4* (herein referred to as *JO-AB-Gal4* ), which contains the same transposon as *JO15-Gal4* (Sharma et al., 2002) but mobilized from the third to the second chromosome (Kay et al., 2016) . A strain containing both *JO15-Gal4* and *JO15-2-Gal4* is herein called *2xJO-AB-Gal4* . To drive expression in the wind-gravity subset of JO neurons, we used *JO31-Gal4* or *JO32-Gal4* (Kamikouchi et al., 2006) which are enhancer traps inserted into the same gene and gave indistinguishable results in our experiments; we pooled the results under the label ( *JO-CE-Gal4* ). The *Lim1* RNAi line, y ^1^ sc ^*^ v ^1^ sev ^21^ ; P{y ^+t7.7^ v ^+t1.8^ =TRiP.HMC03403}attP2 , was obtained from the Bloomington Drosophila Stock Center (BDSC stock 51831). To generate MARCM clones, we used the *Lim1 ^E9^ P{ry ^+t7.2^ =neoFRT}19A/FM7c; P{w ^+mW.hs^ =GawB}GH146 P{w ^+mC^ =UAS-mCD8::GFP.L}LL5/CyO* (BDSC stock 36499) and *y w tub-Gal80 ey-FLP FRT19A; Act-Gal4 UAS-mCD8-GFP/CyO* (Li et al., 2016) strains.

***Antibody Staining & Imaging***

*Drosophila* pupal heads or 3 ^rd^ instar larval eye-antennal disks were dissected in cold phosphate-buffered saline (PBS), then fixed in 4% paraformaldehyde (PFA) for 15 minutes. After three washes in PBS with 0.2% Tween-20 (PBT) with rotation over 30 minutes, the samples were blocked with Blocking Buffer (BB; freshwater fish skin gelatin (0.3 %), normal goat serum (10%) and bovine serum albumin (1%) in PBT) for 1 hour with rotation. Primary antibodies diluted in BB included rabbit GFP-488 antibody (1:500; Invitrogen cat# A11122), Guinea Pig dLim1-568 antibody (1:100; gift of Juan Botas), and mouse Futsch mAb22C10 antibody (1:100; DHSB). Primary antibody incubation took place overnight at 4°C with rotation.

The following day, the samples were washed three times in PBT over 30 minutes with rotation, then incubated with secondary antibodies and phalloidin (pupae) or DAPI (larvae) for 2 hours at room temperature with rotation. Secondary antibody diluted in BB (1:500) consisted of rabbit FITC, guinea pig TRITC, mouse-647 (H&L), as well as phalloidin (Alexa Fluor Plus 405 Phalloidin 1:1000) or DAPI (1:1000). Samples underwent two 15 minute washes in PBT with rotation followed by a brief 5 minute wash in PBS. Finally, samples were mounted in Fluoromount (Thermo Fisher Scientific) onto glass slides with 1.5 coverslips and imaged with a Leica STELLARIS 8 confocal microscope, using a 63x objective lens with oil immersion.

***Electrophysiology/statistical analysis***

Sound-evoked potentials (SEPs) were captured using a pair of electrolytically sharpened tungsten recording electrodes (Eberl et al., 2000; Eberl & Kernan, 2011) . The recording electrode was inserted between the first and secondary segments of the antennae, while the reference electrode was inserted into the head cuticle, near the posterior orbital bristle. A computer-generated pulse song was introduced frontally to the fly under near-field conditions.

Signals were subtracted and amplified with a differential amplifier (DAM50, World Precision Instruments) and digitized at 10 kHz (USB-6001, National Instruments). Average response values were measured as the max-min values in an averaged trace from 10 consecutive presentations of the described protocol. SEP data were analyzed with the Welch’s one-way ANOVA and Dunnett’s T3 post-hoc multiple comparisons using GraphPad Prism software.

***Scanning Electron Microscopy (SEM)***

Flies carrying MARCM clones were dehydrated by immersing in ethanol solutions of varying concentrations- 25%, 50%, 75%, 95%, and 100% -for intervals of 20-30 minutes. The 100% ethanol dehydration was repeated a second time. The samples were then placed into 50:50 solution of hexamethyldisilizane (HMDS) and ethanol for 10-15 minutes, followed by two 10–15-minute intervals in 100% HMDS. The specimens were left to dry overnight in a fume hood. The dehydrated flies were mounted on a metal stub using conductive tape, sputtered with gold under vacuum, and imaged using a Hitachi S-4800 SEM.
